# Effects of Buprenorphine on the Memory and Learning Deficit Induced by Methamphetamine Administration in Male Rats

**DOI:** 10.3389/fnbeh.2021.748563

**Published:** 2021-11-23

**Authors:** Farshid Etaee, Arezoo Rezvani-Kamran, Somayeh Komaki, Masoumeh Asadbegi, Nafiseh Faraji, Safoura Raoufi, Mohammad Taheri, Masoumeh Kourosh-Arami, Alireza Komaki

**Affiliations:** ^1^Neurophysiology Research Center, Hamadan University of Medical Sciences, Hamadan, Iran; ^2^Department of Internal Medicine, Texas Tech University Health Sciences Center, Amarillo, TX, United States; ^3^Skull Base Research Center, Loghman Hakim Hospital, Shahid Beheshti University of Medical Sciences, Tehran, Iran; ^4^Department of Neuroscience, School of Advanced Technologies in Medicine, Iran University of Medical Sciences, Tehran, Iran

**Keywords:** methamphetamine, buprenorphine, learning, memory, interaction

## Abstract

Little is known about the effects of methamphetamine (Meth) and buprenorphine (Bup) on memory and learning in rats. The aim of this investigation was to examine the impact of Meth and Bup on memory and learning. Fourteen male Wistar rats weighing 250–300 g were assigned to four groups: Sham, Meth, Bup, and Meth + Bup and were treated for 1 week. Spatial learning and memory, avoidance learning, and locomotion were assessed using the Morris water maze, passive avoidance learning, and open field tests, respectively. Meth and Bup impaired spatial learning and memory in rats. Co-administration of Meth + Bup did not increase the time spent in the target quadrant compared to Meth alone in the MWM. The Bup and Meh + Bup groups were found with an increase in step-through latency (STLr) and a decrease in the time spent in the dark compartment (TDC). Meth and Bup had no effects on locomotor activity in the open field test. Bup showed a beneficial effect on aversive memory. Since Bup demonstrates fewer side effects than other opioid drugs, it may be preferable for the treatment of avoidance memory deficits in patients with Meth addiction.

## Introduction

Methamphetamine (Meth) is an incredibly addictive psychostimulant with devastating effects on the central nervous system (Mori et al., 2006; Melo et al., 2012; Miladi-Gorji et al., 2015). The prevalence of Meth abuse, as an escalating public health issue (Melo et al., 2012), has increased dramatically, reaching epidemic proportions worldwide (Camarasa et al., 2010) over the last 20 years (Moenk and Matuszewich, 2012). The universality of this drug can be due to its low price and simple production in comparison with cocaine or heroin (Macúchová et al., 2013). Long-term Meth abuse is linked to ventricular hypertrophy, cardiomyopathies, premature atherosclerosis, and kidney failure (Melo et al., 2012). Due to the effects of Meth on neuronal excitability, short-term Meth can cause euphoria and increased alertness and also improve motor activity and awareness (Chen et al., 2012). Meth abuse is associated with significant health problems, including psychosis, psychomotor dysfunction, anxiety, and depression. Clinical investigations have identified impaired functions of the striatal and cortical systems (Krasnova et al., 2010), and several cognitive deficiencies after Meth abuse, even years of drug abstinence (Reichel et al., 2014), leading to deficits in attention, learning, episodic memory, information processing, working memory, decision-making, and impulse control. Meth also causes memory deficits concomitant with reducing hippocampal volume and deleterious structural changes within the hippocampus (Krasnova et al., 2010; Moenk and Matuszewich, 2012; Braren et al., 2014). It diminishes cognition (Melo et al., 2012) and causes significant impairment in the performance of hippocampus-dependent spatial tasks, such as the Morris water maze (MWM) (Vorhees et al., 2000; Ghazvini et al., 2016). Investigations on human fetuses exposed to Meth also have demonstrated cognitive deficits and malformations in different structures of the brain involved in learning (Moenk and Matuszewich, 2012). Some studies have shown that high doses of Meth impaired spatial learning and memory, while lower doses did not (Chen et al., 2012).

Opioids can change the activity of dopaminergic neurons and consequently, alter the pharmacodynamic effect of Meth on the dopaminergic system. It has been reported that the opioid system may be affected by the conditioned cues following Meth administration (Lelong-Boulouard et al., 2006).

Buprenorphine (Bup) is a semi-synthetic partial agonist of the μ-opioid receptor, which has antagonistic effects on the κ- and δ- opioid receptors. It also has poor euphoric impacts and a weak negative effect on respiration (Pournaghash and Riley, 1993; Lelong-Boulouard et al., 2006). Bup causes a limited degree of physical dependency; therefore, it produces better clinical outcomes than morphine and other related agonists (Glover and Davis, 2008). It has been reported that Bup attenuates Meth-induced self-injurious behavior (Mori et al., 2006). Moreover, μ-opioid receptor-mediated pathways may influence the genetic risk for Meth consumption and Bup interferes with the acquisition of Meth intake in mice, potentially due to Bup’s partial agonist activity (Eastwood and Phillips, 2014). Adding Bup to the matrix method resulted in a significant reduction in Meth cravings (Salehi et al., 2015).

Little is known on the effects of Bup on memory function and the reports in this regard are often controversial. The effect of Bup on the improvement or impairment of learning and memory has been evaluated using various tests, experimental species, and doses (Lelong-Boulouard et al., 2006). This investigation was done to examine the efficiency of Bup in inhibiting the learning and memory deficits caused by the administration of Meth in rats.

## Materials and Methods

### Animals

Adult male Wistar rats weighing 250–300 g were studied for the prevention of sex-dependent factors in the present investigation. The rats were randomly categorized into four groups (10 animals per group with computer-generated random numbers) and kept under 12-h dark/light cycles (lights on at 8:00 AM) in a temperature-controlled (22 ± 2°C) colony room. Three days before the tests, animals were housed in considered cages (one rat per cage). All rats had unrestricted access to tap water and food. The researchers responsible for testing the rats were blind to the treatment groups. The rats used in this investigation were treated according to the Guide for the Care and Use of Laboratory Animals and the investigation protocols were proved by the Institutional Animal Care and Use Committee of Hamadan University of Medical Sciences.

### Drugs

The doses of drugs were chosen based on the most of previous studies. Methamphetamine hydrochloride (received from the Presidency Drug Control Headquarters, Tehran, Iran) was dissolved in 0.9% saline (Polston et al., 2011; Melo et al., 2012) and administered at a dose of 2 mg/kg (Lee et al., 2011; Chiang et al., 2014; Miladi-Gorji et al., 2015; Etaee et al., 2017). Bup (FARAN Shimi, Iran) was dissolved in 0.9% saline (Wala and Holtman, 2011) and administered at the dose of 5 mg/kg (Martin et al., 2001; Thompson et al., 2006; Etaee et al., 2017).

### Experimental Design

The routes of drugs administration and the number of days for administration were chosen based on previous studies. The times for the administration of drugs before the tests were chosen based on the time to reach the peak plasma concentration. In this study, 40 rats were divided into the four following groups: Bup: Bup was administered intragastrically or by gavage (IG) (Martin et al., 2001; Thompson et al., 2006) at 5 mg/kg once a day for 7 days (Wala and Holtman, 2011); Meth: Meth was administered intraperitoneally (IP) (Chiang et al., 2014) at 2 mg/kg once a day (Chiang et al., 2014; Miladi-Gorji et al., 2015) for 7 days (Jia et al., 2008); Bup + Meth: Meth and Bup were administered once a day for 7 days; and Sham: Saline was given intragastrically (IG) once a day for 7 days. On the 7th day, Meth and Bup were given to the groups 30 (Schutová et al., 2009) and 60 (Wala and Holtman, 2011) min before testing, respectively.

**FIGURE 1:**
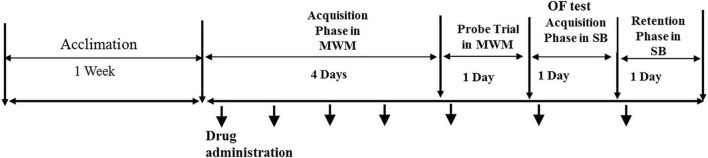
The experimental timeline.

The experimental timeline is demonstrated in Figure 1.

### Morris Water Maze Task

The rats’ learning and spatial memory were assessed using the MWM task, which consisted of a black-colored circular pool (60 cm in height, 180 cm in diameter), filled to a depth of 25 cm with water (22 ± 1°C) (Krasnova et al., 2010; Asadbegi et al., 2017). A dim light was applied to illuminate the area, and the room was soundproof. Several visual cues were present. The pool had four quadrants with four starting lines, named as the north, south, east, and west, and an invisible Plexiglas platform (10 cm in diameter) was positioned centrally 1 cm below the water’s surface in the northwest (target) quadrant (Camarasa et al., 2010).

The training was performed around the same time each day for 4 days, and two blocks of four trials (60 s) were performed per day. A 30-s gap separated the two trials and the rats were allowed to rest for 5 min between the sequential blocks (Jia et al., 2008). During the eight trials, each starting position was randomly used twice. Facing the wall, the rat was positioned in the water at a starting point in one of the four quadrants. Afterward, each rat was allowed to swim in the pool for a period of 60 s to find the hidden platform in the target position. After mounting the platform, the animal was allowed to remain there for 20 s. Therefore, each swim trial took 60 s, the animal was left 20 s on the platform, and 30 s took before the animal was released again.

A video camera, which was positioned above the pool and directly connected to a computer, recorded the rats’ behaviors. The camera recorded the parameters, such as escape latency (the time spent to reach the hidden platform), traveled distance (the length of the swim path to find the hidden platform), and mean speed (the ratio of traveled distance to escape latency) in each trial (Zheng et al., 2002).

One day after the spatial acquisition phase (on day 5), in the retention phase, a probe trial was conducted, in which the platform was removed from the pool, and the rat was allowed to swim for 60 s while we recorded the time spent in the target quadrant (Zarrinkalam et al., 2018). The obtained data were analyzed using Maze Router homemade software.

### Passive Avoidance Test

#### Passive Avoidance Apparatus

In the passive avoidance test, the rats’ natural tendency for a dark environment is used. The device consisted of two chambers that each had a steel-rod grid floor (10 mm apart, 3 mm in diameter; Borj Sanat Co.). One of the chambers (20 × 20 × 30 cm) was provided with a 20 W lamp that was placed centrally at the height of 50 cm, while another chamber was dark with identical size. A guillotine gate (20 × 15 cm) connected the chambers. A dark room was used during the trial session. In the training trial, the guillotine door between the light and dark chambers was closed. When each animal was located in the light chamber with its back to the guillotine door, the door was opened and the time until the rat entered the dark chamber (step-through latency, STLa) was measured with a stopwatch. Then, when the rat entered the dark chamber, the door was closed (Barzegar et al., 2015).

#### Passive Avoidance Training

This test lasted 2 days. On the initial day, all rats were habituated to the device. Each animal was located in the lighted chamber with its back to the guillotine door and 5 s later, the guillotine door was opened (Shiri et al., 2016). Upon entrance to the dark chamber, the door was closed, and the animal was taken from the dark chamber and transferred to the home cage. The habituation trial was replicated after 30 min. The acquisition trial was conducted 30 min later, in which the guillotine door was closed, and a constant current shock (50 Hz, 1.2 mA) (Moradkhani et al., 2015) was applied for 2 s (Khodamoradi et al., 2015; Ganji et al., 2017) immediately after the rat entered the dark chamber. The rat was remained in the device and experienced a foot shock every time it returned to the dark chamber. The training was stopped when the rat remained in the light chamber for 120 s. The number of trials until acquisition (entries into the dark chamber) was recorded. This training was conducted 6 days after drug administration (Motamedi et al., 2010; Zarrindast et al., 2013).

#### Retention Test

The retention experiment was conducted 24 h after the passive avoidance training, and the animal was located in the lighted chamber and 15 s later, the guillotine door was raised (Zarrinkalam et al., 2016). The latency to enter the dark chamber (step-through latency, STLr) and the time spent in the dark chamber (TDC) were recorded (Barzegar et al., 2015). This test was conducted 7 days after onset of drug administration (Jia et al., 2008).

### Open Field Test

Locomotor activity was measured using an open field apparatus, which was made of white acrylic with a size of 50 cm (length) × 50 cm (width) × 38 cm (height). Ambient, low-level, room lights lighted the field. An aloft video camera videotaped the time spent by the animal in the central and peripheral zones, and the data were analyzed with video-tracking software. The rats’ locomotor activity was examined to rule out the probability of differentiation in their baseline activity levels that influenced their performance. The rat was placed in the center of the trial chamber in the open field arena and could explore it for 10 min. The test was conducted for 10 min, 6 days after onset of drug administration (Hollais et al., 2014; Castel et al., 2018).

### Statistical Analysis

The analyses were done using the SPSS version 20.0 (SPSS, Chicago, IL, United States). Statistical significance regarding escape latency traveled distance, and speed in the MWM was assessed using two-way analysis of variance (ANOVA), followed by Bonferroni *post hoc* test. The statistical significance of the results of the open field and passive avoidance tasks and also the time spent in the target quadrant of MWM were compared by the one-way ANOVA, followed by a Tukey’s *post hoc* test. All results were displayed as the mean ± SEM. A *P* < 0.05 was considered statistically significant.

## Results

### Morris Water Maze

#### Escape Latency

**FIGURE 2:**
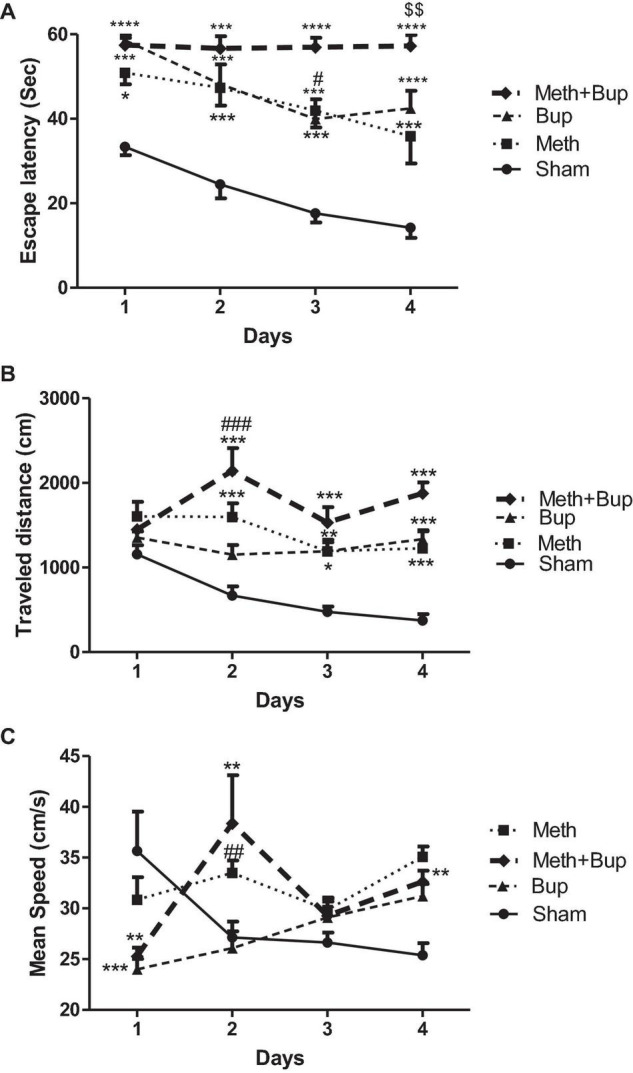
The effect of Bup (5 mg/kg; IG) and Meth (2 mg/kg; IP) on the escape latency **(A)**, traveled distance **(B)**, and mean speed **(C)**, in consecutive training days in the Morris water maze. *P < 0.05, **P < 0.01, ***P < 0.001, ****P < 0.0001: Sham vs. other three groups; ^#^*P* < 0.05, ^##^*P* < 0.01, ^###^*P* < 0.001: Meth + Bup group vs. the Bup group; ^$$^*P* < 0.01: Meth + Bup group vs. the Meth group.

The results of two-way ANOVA showed a significant effect of the drug (*P* < 0.0001), day (*P* < 0.0001), but not their interaction (*P* = 0.21) on the escape latency between groups. Post-test comparisons demonstrated a significant increase in the escape latency after the administration of Meth and Bup or their co-administration in comparison with the Sham group. Our results indicated that the co-administration of Meth + Bup increased the escape latency in comparison with the Meth group on the fourth day of training (*P* < 0.01). It means that the Meth + Bup co-administration accentuated the deteriorating effect of Meth on spatial memory. Moreover, on the third day of training, co-administration of Meth + Bup increased the escape latency in comparison with the Bup group (*P* < 0.05). There were no significant differences between the other groups (*P* > 0.05) (Figure 2A).

Rats in the Sham group showed a significant decrease in the escape latency on the third and fourth days compared to the first day of training (one-way ANOVA; *P* < 0.05 and *P* < 0.01, respectively). In the Bup group, significant reductions in escape latency were observed between the first day of trials in comparison with the third and fourth days (*P* < 0.05). No significant changes were observed in the escape latency of the other groups during training days (*P* > 0.05). Therefore, except for the Sham and Bup groups, other groups did not learn the location of the platform during the training in the MWM (performance was roughly the same during training days).

#### Traveled Distance

The results of two-way ANOVA showed the significant effect of the drug (*P* < 0.0001), day (*P* = 0.0067), and their interaction (*P* = 0.0012) on the traveled distance. Post-test comparisons revealed a significant increase in the traveled distance after the administration of Meth and Bup and their co-administration in comparison with the Sham group. Our results indicated that the co-administration of Meth + Bup on the second day of training increased the traveled distance in comparison with the Bup group (*P* < 0.001). Co-administration of Meth + Bup did not lead to significant changes in the traveled distance in comparison with the Meth group (*P* > 0.05). There were not any significant differences between the other groups (*P* > 0.05) (Figure 2B).

Rats in the Sham group showed a significant decrease in the traveled distance during training days (one-way ANOVA; *P* < 0.001). There was a significant difference in the traveled distance between the first and the second days of training following the co-administration of Meth + Bup (one-way ANOVA; *P* < 0.05). There were no significant differences between other groups in the traveled distance during training days (*P* > 0.05).

#### Mean Speed

The results of two-way ANOVA showed the significant effect of the drug (*P* < 0.001), drug and day interaction (*P* < 0.0001), but not day (*P* = 0.11) on mean speed. A significant difference was observed between the Meth and Sham groups on the fourth day of training in terms of mean speed (*P* < 0.01).

Meth + Bup administration decreased the mean speed in comparison with the Bup group on the second day of training (*P* < 0.001). No significant changes were observed between the other groups (*P* > 0.05 for all) (Figure 2C). There was a significant difference between the first and fourth days (*P* < 0.05). Also, no significant changes were observed between other trial days (*P* > 0.05 for all).

#### Time Spent in the Target Quadrant

**FIGURE 3:**
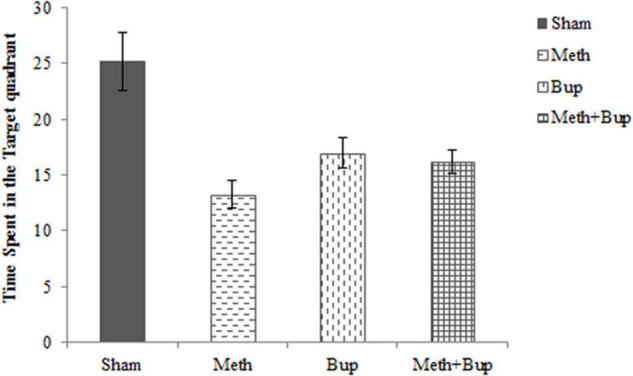
The effect of Bup (5 mg/kg; IG) and Meth (2 mg/kg; IP) on the time spent in the target quadrant in absence of platform in the Morris water maze. Comparisons were made using one-way ANOVA, followed by Tukey’s *post hoc* test. This figure shows that spatial memory deteriorated in the Meth, Bup, and Meth + Bup groups in comparison with the Sham group. *P < 0.05: Bup group vs. the Sham group; **P < 0.01: Meth + Bup group vs. the Sham group; ***P < 0.001: Meth group vs. the Sham group.

The effects of Bup, Meth, and Meth + Bup on the time spent in the target quadrant are shown in Figure 3. Tukey’s *post hoc* test revealed a significant reduction in the time spent in the target quadrant after the administration of Meth (mean ± SEM = 13.24 ± 1.25 s) in comparison with the Sham group (25.22 ± 2.58 s) (*P* < 0.001). Bup administration (16.95 ± 1.37 s) significantly reduced the time spent in the target quadrant in comparison with the Sham group (*P* < 0.05). Also, the co-administration of Meth + Bup (16.18 ± 1.09 s) decreased the time spent in the target quadrant in comparison with the Sham group (*P* < 0.01) (Figure 3).

#### Passive Avoidance Learning

##### Step-Through Latency in the Training Trial

**FIGURE 4:**
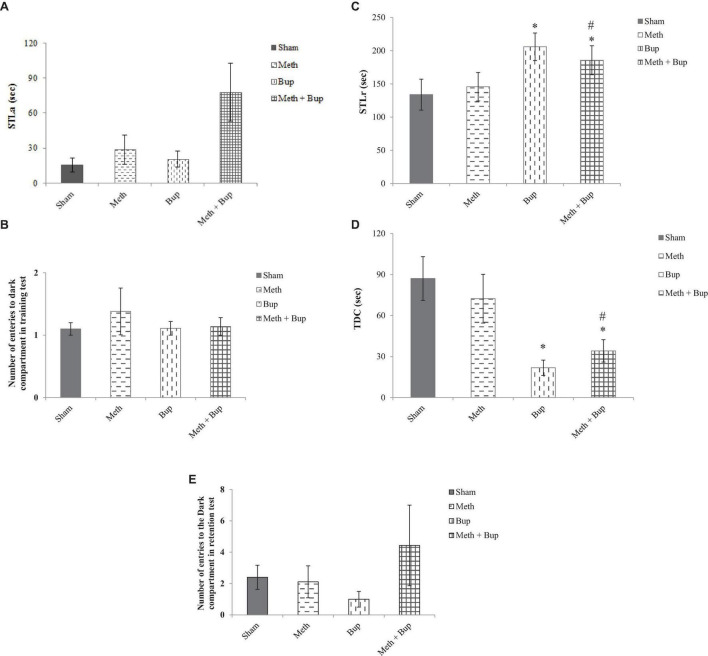
The effect of Bup (5 mg/kg; IG) and Meth (2 mg/kg; IP) on step-through latency in the training test (STLa) **(A)**, trials to acquisition **(B)**, step-through latency in the retention test (STLr) **(C)**, the time spent in the dark compartment (TDC) **(D)**, and the number of entries into the dark compartment **(E)** in the passive avoidance test. *P < 0.05: Bup and Meth + Bup groups in comparison with the Sham and Meth groups; ^#^*P* < 0.05: Meth + Bup group vs. the Meth group.

There were no significant differences in the STLa between the Meth (28.63 ± 12.53 s), Bup (20.67 ± 6.86 s), Meth + Bup (78 ± 24.7 s), and Sham (15.4 ± 5.75 s) group (*P* > 0.05 for all) (Figure 4A).

Regarding the number of entries into the dark compartment during acquisition and training, there were no significant differences between the Meth (1.38 ± 0.375), Bup (1.11 ± 0.11), Meth + Bup (1.14 ± 0.14), and Sham (1.1 ± 0.1) groups (*P* > 0.05 for all) (Figure 4B).

##### Step-Through Latency in the Retention Test

Rats in the Bup (206 ± 20.73 s) and Meth + Bup (185.71 ± 21.8 s) groups showed a significant increase in STLr in the retention test in comparison with the Sham group (133.9 ± 23.3 s) (*P* < 0.05 for both). Co-administration of Meth + Bup significantly increased the STLr in comparison with the Meth group (145.78 ± 21.45 s) (*P* < 0.05) (Figure 4C). STLr was increased in both Bup and Meth + Bup groups. Therefore, the increase in STLr in the Meth + Bup group may be attributed to the effect of Bup. It can be concluded that Bup showed a beneficial effect on aversive memory.

##### Time Spent in the Dark Compartment

Animals in the Bup (21.78 ± 5.62 s) and Meth + Bup (34.14 ± 8.14 s) groups showed a significant decrease in TDC in comparison with the Sham group (87.1 ± 15.95 s) (*P* < 0.05 for both). Co-administration of Meth + Bup significantly decreased the TDC in comparison with the Meth group (72.33 ± 17.82 s) (*P* < 0.05; Figure 4D). Increased TDC in the Bup and Meth + Bup groups suggested and confirmed that Bup (not its co-administration with Meth) exerted a beneficial effect on avoidance memory.

##### Number of Entries Into the Dark Compartment in the Retention Test

Regarding the number of entries into the dark compartment, there were no significant differences between the Meth (2.11 ± 1.02), Bup (1 ± 0.5), Meth + Bup (4.43 ± 2.57), and sham (2.4 ± 0.77) groups (*P* > 0.05 for all) (Figure 4E).

### Open Field Test

#### Locomotor Activity

**FIGURE 5:**
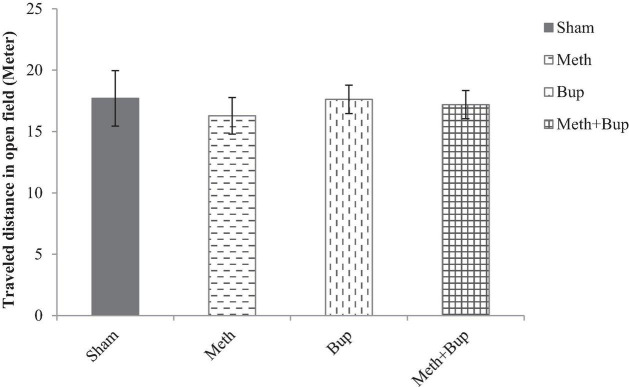
The effect of Bup (5 mg/kg; IG) and Meth (2 mg/kg; IP) on locomotor activity [traveled distance (m)] in the open field test. There were no significant differences between the Meth, Bup, and Meth + Bup groups and the Sham group.

Locomotor activity (traveled distance) was not significantly changed by the administration of Meth (16.28 ± 1.49), Bup (17.62 ± 1.16 m), or Meth + Bup (17.19 ± 1.15 m) in comparison with the sham group (17.70 ± 2.26 m) (*P* > 0.05) or between the groups (*P* > 0.05 for all) (Figure 5).

## Discussion

The results of the current study indicated that Meth, Bup, and Meth + Bup increased the escape latency and traveled distance compared to the sham group, indicating deterioration in the spatial memory. Moreover, Bup decreased the escape latency on the third and fourth days compared to the first day of training. Thus, during training Bup administration alone had only a moderate effect on spatial impairment.

Also, STL and TDC were found to be decreased in the Bup and Meth + Bup groups, indicating an enhancement in the aversive memory. This observation suggests that Bup alone and in combination with Meth exert its positive effects on aversive memory. Therefore, the most interesting finding of the passive avoidance task was the effectiveness of Bup in the potentiation of aversive memory.

The role of Meth abuse in cognitive-related deterioration is not well known. Nevertheless, previous investigations have recommended that while the acute use of Meth may enhance memory and attention, chronic use results in reduced memory function (Seminerio et al., 2013). It has been reported that exposure to Meth during preadolescence improves spatial learning in male rats (Macúchová et al., 2013). Surprisingly, clinical studies have also declared that Meth can result in improved learning and memory function, including visuospatial perception and response speed following limited stimulant usage (Braren et al., 2014). Conversely, repeated Meth injection showed learning and mnemonic impairments (Jia et al., 2008). Some studies have shown that single-day regimens produce cognitive deficits (Braren et al., 2014). Furthermore, repeated Meth-provoked behavioral sensitization caused cognitive impairment. In addition, long-term memory deficiencies have been recognized in chronic Meth abusers (Kamei et al., 2006). Acute administration of a high dose of Meth (>2–10 mg/kg) caused impairments in spatial and non-spatial memory functions, caused by the degeneration of serotonergic and dopaminergic nerve terminals in the brain (Lee et al., 2011). Accordingly, animals previously exposed to a 1-day Meth regimen had dysfunctions in an appetitive maze sequential learning task and motor performance tasks and demonstrated mild spatial memory dysfunctions (Marshall et al., 2007). In contrast, low-dose Meth (2 mg/kg) injection caused hyperlocomotion but did not alter memory (Lee et al., 2011). The importance of morphine and other opioids in memory processes has been well described (Lelong-Boulouard et al., 2006).

Our data demonstrated that the co-administration of Meth + Bup increased the escape latency in comparison with the Meth group on the fourth day of training. It means that Bup partially potentiated Meth-induced memory impairment. In agreement with our results, it has been shown that opioid receptor agonists may diminish short- and/or long-term memory processes (Lelong-Boulouard et al., 2006). In this regard, the endogenous opioid system may play a crucial role in stress-induced memory impairment (Cao et al., 2015). In addition, Bup induced deficits in long-term memory in the passive avoidance test and short-term memory deficits in the Y-maze test (Lelong-Boulouard et al., 2006). Stimulation of κ-opioid receptors enhanced memory dysfunction arising from the blockade of muscarinic M1 receptors (Ukai et al., 1997). In contrast, acute administration of Bup improved short-term memory for social reward cues (Syal et al., 2015).

After repeated administration, Meth adheres to the dopamine transporter (DAT) on dopaminergic nerve terminals through a pseudo-transmitter function, which increases dopamine release but represses dopamine reuptake, leading to an increase in the level of dopamine in the synaptic cleft (Chen et al., 2012; Seminerio et al., 2013). As a result, Meth triggers dopamine release from the cytosol within the extracellular space using reverse transport by the DAT (Melo et al., 2012). Consistently, acute treatment with a low dose of Meth (2 mg/kg) enhanced striatal extracellular concentrations of dopamine. Furthermore, Meth reduced the intraneuronal oxidative metabolism of dopamine by the repression of monoamine oxidase (Pereira et al., 2011). Also, acute administration of Meth caused an increase in the dopamine level in the nucleus accumbens (Macúchová et al., 2013).

Meth is neurotoxic and directly damages neurons and memory function (Chen et al., 2012). Dopamine pathways are involved in cognition (Macúchová et al., 2013). Dopamine has been exhibited to change various cognitive functions, including attention, memory, response inhibition, and task switching (Seminerio et al., 2013). Long-term use of Meth damages dopaminergic systems and reduces various indices of dopamine terminal integrity, particularly in the striatum (Camarasa et al., 2010; Braren et al., 2014). Meth affects both the prefrontal cortex and striatum via the dopaminergic system. Dopamine deficiencies in the striatum decrease simple task performance and reaction time while dopamine deficits in the prefrontal cortex contribute to cognitive dysfunction (Seminerio et al., 2013). Changes caused by chronic exposure to Meth in rats include selective damage to dopaminergic terminals within the dorsal striatum and hippocampus, long-lasting decreases in dopamine content, loss of dopamine transporters, and a reduction in the activity of tyrosine hydroxylase and DAT (Camarasa et al., 2010; Braren et al., 2014). Vesicular monoamine transporter 2 has an essential role in Meth-induced toxicity (Melo et al., 2012). Meth reduces long-term potentiation (LTP) and produces synaptic maladaptation through changing excitatory synaptic transmission by the activation of the serotonin and dopamine receptor systems (Chen et al., 2012).

Furthermore, Meth use leads to nerve terminal degeneration by the production of nitrogen and reactive oxygen species. Nerve terminal and axonal degeneration can occur in the nigrostriatal dopaminergic projections, potentially resulting in cognitive impairment (Seminerio et al., 2013). Meth also induces cortical cell degeneration. Specifically, the Meth-induced degeneration of cortical neurons may only express the most apparent consequences of the powerful and enduring effects of Meth on the cerebral cortex (Marshall et al., 2007). This evidence is furthermore confirmed by the reports of the positive association between Meth intake and cell death in the prefrontal cortex and negative association between Meth intake and hippocampal volume in rodents (Krasnova et al., 2010).

Bup causes a progressive increase in extracellular dopamine and enhances basal levels of dopamine in the NAc (Sorge and Stewart, 2006). Also, Bup, like other partial μ-receptor agonists, stimulates the release of dopamine in the mesolimbic system (Nantwi et al., 1998). Moreover, opioid receptor agonists can change the activity of dopamine neurons and alter the pharmacodynamic impacts of Meth on the dopaminergic system (Sorge and Stewart, 2006).

Meth increases glutamate release in various brain regions, such as the cerebral cortex, hippocampus, and striatum (Lee et al., 2011). Conversely, prolonged administration of Bup can decrease glutamate function. Continuous exposure to Bup also results in reduced glutamatergic activity in the NAc and striatum (Sorge and Stewart, 2006). In contrast, Bup treatment increased basal levels of glutamate in the NAc of rats (Placenza et al., 2008). The glutamatergic system is perturbed by Meth exposure, leading to increased glutamate signaling. GluA2 (GluR2) incorporation into AMPA receptors in multiple brain regions is associated with Meth treatment and subsequent reduction in glutamate overflow and associated excitotoxicity (Olsen et al., 2013). In addition, Meth-induced impaired memory function due to alterations of N-Methyl-D-aspartate (NMDA) receptor binding sites in the hippocampus and prefrontal cortex may also underlie the memory and learning deficits correlated with the administration of it (Lee et al., 2011). NMDA receptors are needed for synaptic plasticity associated with learning and memory (Lee et al., 2011; Barzegar et al., 2015).

Other neurotransmitter systems, in addition to the dopaminergic and glutamatergic systems, may be involved in the effects of Meth and Bup on learning and memory. The increased, unchanged, or decreased serotonin (5HT) content after Meth administration has been reported (Melo et al., 2012). Meth may cause a decrease in the concentration of 5HT and its particular metabolites, a reduction in the number of 5HT transporter binding sites, a decline in the activity of tryptophan hydroxylase, and the loss of 5HT transporters in the striatum and hippocampus (Marshall et al., 2007). Conversely, opioids can increase the 5HT release and their analgesic effect depends partly on the consequent activation of postsynaptic 5HT1_A_ receptors (Tao et al., 2003). Gamma-aminobutyric acid (GABA) receptors play a role in Meth-associated rewarding memories (Jiao et al., 2016). Intra-NAc infusion of muscimol (a GABA receptor agonist) reduced Meth-induced enhancement of LTP in the dentate gyrus-, while the infusion of AP5 (an NMDA receptor antagonist) inhibited Meth-induced improvement of LTP (Heysieattalab et al., 2016). The existence of interactions between GABAergic and opiate systems in the brain has been declared. The extensive concentration of opioid receptors in the limbic system (thalamus, amygdala, and NAc), hippocampus, and cerebral cortex suggests that GABA neurons are involved in emotional responses and many other cognitive processes (Lelong-Boulouard et al., 2006). In addition, the opioid-induced release of dopamine is likely a secondary response, owing to the inhibition of GABA interneurons, which results in the disinhibition of dopaminergic neurons (Johnson and North, 1992).

## Conclusion

In summary, this study demonstrated that Bup administration accentuates the learning and memory impairment induced by Meth administration in rats. While Bup impairs spatial learning and memory, it potentiates aversive memory. Since Bup exhibits lower side effects compared to other opioids, it may be desirable for the treatment of avoidance memory discrepancies in patients with Meth addiction.

## Data Availability Statement

The original contributions presented in the study are included in the article/supplementary material, further inquiries can be directed to the corresponding author/s.

## Ethics Statement

Rats used in this investigation were treated under the Guide for the Care and Use of Laboratory Animals, and the investigation protocols were proved by the Institutional Animal Care and Use Committee at Hamadan University of Medical Sciences (IR.UMSHA.REC.1400.346).

## Author Contributions

MT, AK, and FE wrote the manuscript and revised it. AR-K, NF, and SK supervised the study and performed the experiment. MA and SR analyzed the data. All authors approved the manuscript.

## Conflict of Interest

The authors declare that the research was conducted in the absence of any commercial or financial relationships that could be construed as a potential conflict of interest.

## Publisher’s Note

All claims expressed in this article are solely those of the authors and do not necessarily represent those of their affiliated organizations, or those of the publisher, the editors and the reviewers. Any product that may be evaluated in this article, or claim that may be made by its manufacturer, is not guaranteed or endorsed by the publisher.
